# Early muscle hypotonia as a potential marker for autism spectrum disorder: a systematic review

**DOI:** 10.3389/fpsyt.2025.1598182

**Published:** 2025-09-18

**Authors:** Ting Zhang, Jinying Wang, Zhenkun Cao, Yuhan Ma, Zhihai Lv

**Affiliations:** ^1^ Jiamusi University School of Rehabilitation Medicine, Jiamusi, Heilongjiang, China; ^2^ Longgang District Maternity & Child Healthcare Hospital of Shenzhen City (Affiliated Shenzhen Women and Children’s Hospital (Longgang) of Shantou University Medical College), Shenzhen, Guangdong, China; ^3^ Georg-August, University of Göttingen, Faculty of Social Sciences, Göttingen, Germany

**Keywords:** autism spectrum disorder, hypotonia, motor skills, child development, interfere

## Abstract

**Background:**

The diagnosis of ASD has increased globally owing to the expansion of diagnostic criteria, increased awareness, and improvement in symptom identification. However, the diagnosis of ASD in young or neurodivergent people remains challenging and requires the investigation of new early indications.

**Objectives:**

In this review, we examined the correlation between early hypotonia (including motor difficulties) and ASD, evaluating the potential of hypotonia as an early biomarker and screening instrument.

**Methods:**

Using the PRISMA criteria (PROSPERO: CRD42024626398), we searched PubMed, Embase, Cochrane Library, and Web of Science without any constraints on date or language. The inclusion criteria were derived from studies on children aged 0–6 years that investigated hypotonia (e.g., motor impairments or head lag) in connection with ASD diagnosis or characteristics. The eligible studies were prospective cohort, case-control, and retrospective video-analysis studies. Two researchers independently collected and evaluated the data.

**Results:**

Twenty-four studies (prospective cohort, case-control, or video analyses) were included in this review.The participants were aged 2 months to 6 years and included infant siblings of autistic children (a cohort with elevated likelihood of an autism diagnosis), children with familial ASD, and individuals from the general population.The research showed a consistent association of hypotonia and motor difficulties with ASD, despite variations in assessment methodologies, such as standardized motor measures and clinical evaluations. However, despite methodological heterogeneity, cumulative evidence supported the potential of hypotonia as an early ASD biomarker.

**Conclusion:**

Hypotonia and related motor differences may serve as practical screening indicators of increased likelihood of a later autism diagnosis. Identifying these signs can prompt earlier referral and support. While the findings are promising, further research is needed to standardize assessment protocols and validate clinical utility. Interdisciplinary collaboration may facilitate early detection, enhancing long-term outcomes through timely assistance.

**Systematic review registration:**

https://www.crd.york.ac.uk/PROSPERO/myprospero, identifier CRD42024626398.

## 1 Introduction

Autism spectrum disorder (ASD) is a neurodevelopmental condition marked by enduring impairments in social communication and interaction alongside restricted and repetitive behavior, interests, or activities (1). In recent years, the age for autism diagnosis has decreased owing to the progressive broadening of diagnostic criteria and an increase in public awareness and sensitivity to mild autism characteristics (2, 3). The global prevalence of autism continues to increase (4). While clinicians can establish a potentially accurate diagnosis once the child enters their second year (5, 6), numerous problems persist in early autism diagnosis, particularly in younger children or atypical cases (7).

Studies have shown that the earlier abnormal development is identified and detected, the earlier the patient can undergo treatment, which can help improve the condition and reduce the burden on the family, especially during the critical period of neurodevelopment. Early intervention not only significantly improves a child’s social communication, behavioral regulation, and adaptability but also significantly improves long-term prognosis by optimizing brain function at the critical stage of neural connectivity formation (1, 8). Therefore, exploring new early markers to optimize autism screening strategies is a central issue in current research.

A recent study shows that 71.5% of 8-year-old autistic children met criteria for motor milestone delays (9). Hypotonia is a prevalent clinical characteristic in autistic children with motor milestone delays and affects approximately 32.3% of the population( (10). Furthermore, hypotonia is significantly correlated with other autism-related clinical characteristics, such as impairments in social interaction and delayed language acquisition (10–12). This prompts a thorough examination of the possible role of hypotonia as an early screening tool for autism.

In this systematic review, we attempted to comprehensively analyze data from published studies to evaluate the potential use of hypotonia in early screening for autism and offer fresh insights into early identification procedures. We focused not only on the potential of hypotonia as an early indicator of autism but also consolidated findings from related studies to validate the results and assess the practicality of clinical application, particularly in establishing a robust foundation for guiding early intervention efforts. Considering the inherent limitations of retrospective investigations—such as potential biases in historical records and recollections—we selected studies with retrospective video data for our analysis. In such studies, the behavioral patterns of infants and toddlers are coded and analyzed by researchers to provide significant insight into the correlation between hypotonia and autism traits. By expanding the inclusion criteria, we attempted to deliver a more thorough and systematic overview of existing research in this domain to guide future investigations and, eventually, enhance the precision and promptness of early autism screening. These findings can aid early intervention and evaluation for a larger number of children at elevated likelihood of a later autism diagnosis.

## 2 Methods

### 2.1 Agreement and registration

The research procedure for this systematic review was registered with the International Platform for the Registration of Systematic Reviews (registration number: PROSPEROCRD42024626398) and adhered to the requirements outlined in Preferred Reporting Items for Systematic Reviews and Meta-Analyses (PRISMA) (13).

### 2.2 Inclusion and exclusion criteria

To conduct the review, we selected studies using specific criteria. The inclusion criteria were as follows. (1) Studies on children aged 0–6 years, with emphasis on the early developmental phases of newborns and toddlers (0–24 months), or studies on elevated likelihood groups for autism (e.g., siblings of children with autism) or the general population. (2) Studies on hypotonia metrics as potential predictors of autism, including motor difficulties resulting from hypotonia—such as fine motor hypoplasia, head lag, and gait delays—in which scales and motor assessments that specifically evaluate hypotonia characteristics were used. (3) Prospective studies, longitudinal cohort studies, case-control studies, or retrospective video analyses including a subsequent diagnosis or assessment of characteristics related to autism. (4) Studies with a formal diagnosis of autism or a measurement of autism-spectrum-related traits, with precise data to support the association between hypotonia (including motor developmental disorders caused by it, such as head lag and fine motor delay) and autism outcomes. We explored the association between early-onset hypotonia and autism.

The exclusion criteria were as follows: (1) Studies that excluded children with autism or at elevated likelihood for autism, or those that solely included participants with alternative neuromuscular conditions (e.g., cerebral palsy or muscular dystrophy). (2) Studies that failed to measure or explicitly report dystonia-related characteristics or those that solely evaluated other autism-related traits (e.g., social interaction or language development) without addressing dystonia. (3) Studies that focused only on the behavioral characteristics of children with autism, without conducting a comparative analysis of typically developing (TD) or low-likelihood youngsters. (4) Review articles, opinion pieces, editorials, animal experiments, single case studies, and studies that did not provide data on autism diagnosis or related characteristics. (5) Studies with an unclear analysis method, with unreliable conclusions and repeated sample research. (6) Informal published literature, such as manuscripts, dissertations, or conference abstracts that were not peer reviewed. (7) Studies with a sample size of less than 20.

To ensure comprehensiveness in the scope of the study while balancing scientific validity and flexibility, we ensured that the inclusion criteria allowed for the supplementation of indirect studies as supporting evidence to the predominantly direct studies. Eligible studies directly examined the association between hypotonia and the likelihood of a later autism diagnosis and reported extractable data or an explicit conclusion. Indirect studies included those involving early motor abnormalities (e.g., fine motor, grasping behaviors, or postural control), which may indirectly reflect the role of hypotonia. The inferential nature and applicability of the conclusions from indirect studies will be clarified in the Discussion regarding their limitations.

### 2.3 Search strategy

This review followed the PRISMA 2020 Statement (13) and conducted a two-stage systematic search in PubMed, Embase, Cochrane Library, and Web of Science databases. The first stage focused on the “association between hypotonia and autism,” with standardized subject terms (e.g., PubMed’s MeSH terms “Autistic Disorder,” “Muscle HypotoniaMuscle, “EmBase’s EmTree terms “autism,” “muscle hypotonia”) and expanding free words (e.g., “Autism,” “Tree,” “Autism,” “Autism,” “Muscle Tree”). “Autism,” “Muscle Tone Atonics.” The study types were restricted to Cross-Sectional Studies, Randomized Controlled Trials, Cohort Studies, and Prospective Studies. An example of the search formula is as follows: (Autistic Disorder OR autism OR “autism spectrum disorder”) AND (hypotonia OR “Muscle Tone Atonics”) AND (Cross-Sectional Studies OR Randomized Controlled Trials OR Cohort Studies OR Prospective Studies). Phase 2: for “motor association between and autism,” we modified the keywords “Motor Skills” and “Motor Skills Disorder” and their variants (e.g., “Motor Skills Disorder”), as well as “Child Development” and “Developmental Coordination Disorder.” The remaining conditions were consistent with those in Phase 1—stage 1 uniformity. We conducted a preliminary exploratory review of the literature in the pertinent field to verify that the key terms accurately represented the scope and complexity of the study. We subsequently selected the subject terms and keywords based on these findings. We refined the search algorithms using the database thesaurus and integrated Boolean operators (AND/OR) within the title, abstract, and keyword fields for synonym retrieval. Additionally, we imposed no limitations on the publication date or language for the search results. The search results were not limited by the publication date or language. After preliminary screening, the researchers removed duplicates using EndNote. They manually searched for additional pertinent literature by examining the references of numerous seminal publications and using Google.com to identify literature analogous to the included studies.

## 3 Results

### 3.1 Screening and inclusion

EndNote X9 was used to de-duplicate data from the selected studies by eliminating duplicate records based on precise information matches, including author names, title, year of publication, publication, and volume issue. Subsequently, using EndNote, the first author further verified and eliminated potential duplicate documents identified by authors and titles by the authors through abstract review. Upon completion of de-duplication, the first author conducted a preliminary screening of the literature for relevance, using titles and abstracts to eliminate studies unrelated to the study topic.The authors (ZT and WJY) conducted an anonymous review of the initially screened studies and conferred with the authors (CZK and MYH) about the application of inclusion and exclusion criteria to address potential discrepancies and maintain a high level of consistency in the screening process. To guarantee the inclusion of the latest findings, all databases were re-examined in December 2024. A manual review of the studies published since the last search was conducted, which resulted in the incorporation of 24 qualifying studies (**Table 1**). 

**Table 1:** Summary of the studies included in this review (n=24).

Study	Study design	Participants	Assessment tool	Findings and conclusions	Quality of evidence
Landa et al. (2006) (14)	Prospective research monitoring motor, linguistic, and social competencies in high-risk newborns.	Experimental group: 87 newborns, comprising 60 high-risk infants (siblings of children with Autism Spectrum Disorder); Control group: 27 low-risk infants.	ASD: ADOS Motor development:MSEL	- No differences between groups at six months. - At 14 months, the ASD group had delays in all areas except visual reception compared to the comparison group without an autism diagnosis. - At 24 months, the ASD group had difficulties across all domains compared to the comparison group without an autism diagnosis and in motor and verbal skills relative to the LD group. Reduced developmental progression in Autism Spectrum Disorder.	8/9 High
Bolton et al. (15)	Prospective study evaluating early motor and social communication factors in children.	Experimental group: 86 children subsequently diagnosed with Autism Spectrum Disorder (ASD); Control group: Remaining children in the Avon cohort (n=13,885).	ASD: Parental	Early markers: Social, motor, and communication differences were detectable by 6 months, and vision/hearing concerns emerged in the first year. - Developmental trajectory: Widening gap in social, language, and play skills between ASD and comparison group without an autism diagnosis by 24–30 months (R²=0.42, p <.001). Core difficulties: Social communication impairments (p <.001). - Core difficulties: Social communication impairments (e.g., reduced gestures, delayed vocabulary) strongly predicted ASD (OR=0.31-0.43, p <.001) and broader autistic traits. Clinical relevance: 45% of ASD cases showed worsening social/communication skills between 14 and 24 months.	8/9 High
Flanagan et al. (16)	Prospective study; Pull-to-Sit assessment	High-risk: Siblings of children with Autism Spectrum Disorder (Sample 1: n=40; Sample 2: n=20). Low-risk: Absence of familial history of ASD (Sample 2: n=21).	ASD: ADOS, Motor development:MSEL	Sample 1: - Ninety percent (9/10) of infants subsequently diagnosed with Autism Spectrum Disorder (ASD) demonstrated head lag at six months (p=.020). Example 2: - 75% (15/20) of high-risk newborns compared to 33% (7/21) of low-risk infants had head lag (p=.018). - Head lag linked to the likelihood of Autism Spectrum Disorder and neurodevelopmental impairment.	6/9 Moderate
Landa et al. (17)	Retrospective cohort study with longitudinal follow-up and blinded evaluation.	235 children, aged 6 to 36 months, with and without a sibling diagnosed with autism. Children were categorized as follows: ASD identified by 14 months, ASD identified after 14 months, and no ASD.	ASD: ADOS Motor development:MSEL	Early Autism Spectrum Disorder group. - Expressive language scores at 14 months were markedly lower compared to the Later-ASD group (difference of 3.1 points, p <.001) - Fine motor delays at 36 months compared to the non-ASD group (difference of 2.8 points, p <.01). - Expressive language stagnation between 30 and 36 months (no advancement) Subsequent-ASD cohort. - Diminished consonant inventory at 14 months compared to the Non-ASD group (difference of 0.9 points, p=.005) - Stagnation in social joint attention increases after 24 months (p=.005).	9/9 High
LeBarton et al., (2013) (18)	Longitudinal study of motor skills in infancy predicting ASD diagnosis and language outcomes.	140 infants (89 high-risk, 51 low-risk) assessed from 6 months to 36 months.	ASD: ADOS Motor development:PDMS-2	- Study 1: ASD group showed poorer motor skills at 6 months vs. LR group, particularly in Visual-Motor Integration (ASD: 31.05 ± 1.59 vs. LR: 33.24 ± 0.83; *p=*.032). Stationary (ASD: 27.80 ± 1.49 vs. LR: 29.25 ± 0.55; *p=*.069) and Grasping (ASD: 27.20 ± 0.99 vs. LR: 27.96 ± 0.55; *p=*.082) showed trends. - Study 2: Grasping at 6 months predicted expressive language at 30 months (β=0.75, *p* <.05) and 36 months (β=0.997, *p* <.01). Stationary predicted language at 30 months (β=0.63, *p* <.05).	9/9 High
Lemcke et al. (19)	Prospective study identifying early signs of ASD through national birth cohort data.	Experimental: Children later diagnosed with ASD (n=720); Control: Children with intellectual disability (n=231) and typically developing.	ASD: Maternal interviews, Registry data	At six months. - Motor delays: The ASD group could not maintain an upright sitting position (HR=1.8, p < 0.05); the ID group demonstrated a more significant risk (HR=3.5, p < 0.05). - Diminished interest in object acquisition (ASD: HR=2.4, p < 0.05; ID: HR=11.7, p < 0.05). At eighteen months. - Language delay: 73.4% of children with ASD utilized ≤10 words (HR=2.0, p < 0.05); in the ID group, 86.3% (HR=4.5, p < 0.05). - Social delays: 10.3% of children with ASD were unable to retrieve objects (HR=6.1, p < 0.05); CA subgroup: 20.4% (HR=13.5, p < 0.05). - Motor delays: Children with ASD exhibited delayed ambulation (mean age: 13.7 months compared to cohort: 12.6 months, p < 0.0001).	9/9 High
Libertus et al. (20)	Prospective investigation of fine motor and gripping abilities in high-risk versus low-risk babies for Autism Spectrum Disorder (ASD).	Experiment 1: 129 infants aged 6 months (107 high-risk, 22 low-risk) Experiment 2:42 involved 6-month-old infants, comprising 23 high-risk and 19 low-risk subjects, with 26 participants following up to 10 months of age.	ASD: ADOS-G, Motor development:MSEL	- High-risk newborns exhibited less advanced object manipulation in a structured setting (as assessed by the Mullen Scales of Early Learning) and less grasping activity during free play compared to infants without a familial history of ASD.	9/9 High
Heathcock et al. (21)	Retrospective examination of motor skill development in high-risk versus low-risk babies for Autism Spectrum Disorder.	Experimental: 25 high-risk infants; Control: 14 low-risk infants.	ASD: DSM-IV, Motor development:AIMS	High-risk children exhibited less midline play at 4 months compared to low-risk infants; however, this difference was not statistically significant (p=.29). - AIMS scores: Notable group disparity at 4 months (LR > HR, t(30)=-3.18, p=.003). - ASD subgroup: 3 out of 5 infants with ASD scored below the high-risk mean on the AIMS at 6 months; 4 out of 5 had diminished midline play.	6/9 Moderate
Yoshioka et al. (2015) (22)	Cohort research examining the follow-up of infants diagnosed with hypotonia	Floppy Newborns Group: Comprised of 16 newborns with delayed motor development (hypotonia). The control group had 16 newborns exhibiting usual development and everyday motor skills.	ASD: DSM-IV, DQ	- The investigation focused on infants exhibiting motor development delays and hypotonia, which were identified during health check-ups. Sixteen cases were examined in the 4-month health check cohort and 16 cases in the 9-month health check cohort. Of the newborns exhibiting enhanced muscle tone reduction and walking capability, 19% received a diagnosis of autism spectrum condition. Of the newborns exhibiting enhanced muscle tone reduction and the capacity to walk, 19% received a diagnosis of autism spectrum condition.	6/9 Moderate
Bishop et al. (23)	Cross-sectional study comparing ASD probands with/without *de novo* mutations.	Experimental Group: 112 ASD probands exhibiting *de novo* loss-of-function/copy number variation mutations. Control Group: 112 ASD probands devoid of mutations, matched by sex, age, and nonverbal IQ.	ASD: ADI-R, ADOS, SRS Motor development:ADI-R	- *De novo* mutations associated with “muted” ASD characteristics and early motor delays indicate that motor delays may serve as a possible genetic marker for ASD.	9/9 High
Serdarevic et al. (12)	Longitudinal cohort study based on population	2,905 newborns; evaluation of muscular tone at 0–5 months and assessment of autistic characteristics at 6 years	ASD: SRS, PDP Motor development:Touwen’s Neurodevelopmental Examination	- Reduced baby muscle tone was associated with increased autistic traits: SRS (adjusted β=0.05, 95% CI: 0.00-0.02, 0.04-0.10, P=0.01); PDP (adjusted β=0.08, 95% CI: 0., P < 0.001). 0.04-0.10, p < 0.001. - Clinical PDP (>98th percentile): adjusted odds ratio=1.36 (95% confidence interval: 1.08-1.72, P < 0.01). - No correlation with elevated muscular tone. Results remained consistent following the exclusion of ASD cases.	9/9 High
Boin Choi et al. (24)	Longitudinal study examining fine motor skills and linguistic outcomes in high- and low-risk infants.	170 newborns: 30 high-risk infants subsequently identified with ASD (HRA+), 71 high-risk infants without ASD (HRA-), and 69 low-risk controls (LRC).	ASD: ADOS Motor development: AIMS, Skilled Reaching Rating Scale	Infants with HRA+ had diminished fine motor development (6–24 months) compared to HRA- (β=-0.83, p=0.04) and LRC (β=-0.51, p=0.09, marginal). - Fine motor abilities at 6 months predicted expressive language at 36 months (β=4.45, p < 0.001). Cross-sectional group differences were seen at 12 months (HRA+ vs. HRA-: p=0.015) and 18 months (HRA+ vs. LRC: p=0.02).	9/9 High
Sacrey et al. (25)	Analysis of motor mechanics in babies at risk for Autism Spectrum Disorder (ASD).	- HR-ASD: 10 infants with older ASD siblings, later diagnosed with ASD. - HR-N: 10 infants with older ASD siblings, not diagnosed with ASD. - LR: 10 controls (7 boys, 3 girls) with no ASD family history. Enrolled at 6–12 months.	ASD: AOSI (6–15 months), ADOS (18–36 months). Motor development: AIMS, Skilled Reaching Rating Scale	- HR-ASD exhibited superior overall reach-to-grasp scores compared to LR and HR-N (HR-ASD: 5.17 ± 0.99 vs. LR: 4.15 ± 1.04, p=0.001; HR-N: 4.52 ± 1.12, p=0.033). - Components: HR-ASD exhibited inferior scores in Orientation (0.81 ± 0.26 vs. LR: 0.65 ± 0.26), Lift (1.73 ± 0.48 vs. LR: 1.03 ± 0.50), and other metrics (p < 0.05). Pronate (1.10 ± 0.31 vs. LR: 0.82 ± 0.32) (p < 0.05).	6/9 Moderate
Øien et al. (26)	Longitudinal analysis of children passing 18-month ASD screening later diagnosed with ASD.	68,197 screen-negative children; 228 later diagnosed with ASD (false-negative group).	ASD:M-CHAT 6 critical items (fail <2=screen negative). Motor development: ASQ	- The 228 false-negative ASD children had slower fine motor development than the true-negative children, with an average difference of d=0.399 (p < 0.001). - Girls exhibited larger effect sizes in social (*d=*0.657 vs. 0.303) and gross motor delays (*d=*1.06(L) vs. 0.267(S)).	8/9 High
Serdarevic et al. (27)	Genetic cohort study; polygenic risk scores (PRS) and neuromotor development	Subset of 1,174 infants (aged 9–20 weeks) underwent neuromotor assessments; autistic traits evaluated at age 6. |	ASD: PRS, SRS Motor development. Touwen's Neurodevelopmental Examination	- Higher ASD-PRS associated with less optimal infant neuromotor development (e.g., low muscle tone: β=0.068, *p* =0.01). - ADHD-PRS linked to less optimal sensory responses (β=0.035, *p* =0.02). - ASD-PRS and ADHD-PRS both predicted autistic traits at age 6 (ASD: β=0.08, *p* =0.01; ADHD in boys: β=0.176, *p* < 0.001). - SNP-based heritability: neuromotor development=20% (SE=0.21), autistic traits=68% (SE=0.26). - Genetic correlation between neuromotor development and autistic traits: *r* =0.35 (SE=0.21, *p* < 0.001).	9/9 High
Gabis et al. (11)	Retrospective cohort analysis of developmental milestones in children with ASD.	467 children diagnosed with ASD (111 girls, 24%; 356 boys, 76%)	ASD : ADOS Motor development:MSEL,DDST II	- Motor delay: 60% of girls vs. 47% of boys with ASD (χ²=6.56, p<0.01). - Global Developmental Delay (GDD): 49% of girls vs. 36% of boys (χ²=5.87, p<0.05). - Age of walking: Girls walked later (mean=17.9 months) vs. boys (mean=16 months; *t*(330)=2.43, p<0.05). - Idiopathic ASD: 42.7% of girls vs. 31% of boys had motor delays (χ²=3.84, p=0.05). |The results of this study are summarized in the following table.	6/9 Moderate
Licari et al. (28)	Prospective study of motor difficulties and predictive markers for emerging ASD phenotypes.	Experimental: 96 infants with early ASD behavioral signs; no explicit control group.	ASD : ADOS-T Motor development:MSEL	- It was found that at baseline, 65.6% of the infants had gross motor delays, and 30.2% had fine motor delays. - Lower fine-motor scores at baseline and during follow-up were associated with higher ADOS - T scores (β=-0.12, p=0.01).	8/9 High
Mohd Nordin et al. (29)	Retrospective comparison of motor delays in ASD children vs. typically developing children.	178 children (104 ASD, 74 typically developing); ages 12–60 months.	ASD : DSM-5 Motor development:SGS II	- Motor delay prevalence. - Gross motor delay in ASD: 6.7% (vs. 0% in TD; p=0.02). - Fine motor delay in ASD: 38.5% (vs. 0% in TD; p < 0.01). - Age effect. - Gross motor delay was only observed in older ASD children (37–60 months; 13.2% vs. 0% in 0–36 months; p=0.01). - Fine motor delay increased with age (52.8% in older vs. 23.5% in younger ASD group; p=0.03).	6/9 Moderate
López-Espejo et al. (10)	Cross-sectional study, motor impairments in autistic children	96 autistic children, median age 4 years	ASD: DSM-5, ADOS-2	- 63.5% had motor disturbances. - 33.3% exhibited motor stereotypes (hand/body movements). - 28.1% had delayed independent gait (>16 months corrected age). - 40.6% showed abnormal muscle tone (32.3% hypotonia, 8.3% hypertonia). - Delayed gait was associated with no verbal language at age 4 (OR=9.36; 95% CI=2.67-32.78). - Infant hypotonia predicted delayed gait (OR=2.65; 95% CI=1.08-6.48) and stereotypes (OR=2.63; 95% CI=1.04-6.65).	6/9 Moderate
Patterson et al. (2022) (30)	Prospective study of motor trajectories in infant siblings at high and low risk for ASD.	Experimental: 499 high-risk infants; Control: 176 low-risk infants.	ASD: ADOS, ADI-R Motor development:MSEL	Motor trajectories and ASD diagnosis. - Gross motor: low/stable trajectory (Group 1) had 38.0% EL-ASD vs. 14.9% in the high/stable group (Group 4) (χ²=15.40, p<0.05). - Fine motor: Low/stable trajectory (Group 1) comprised 75.6% EL-ASD vs. 5.0% in high/accelerating group (Group 4) (χ²=153.29, p<0.001). ASD symptom severity. The fine motor low/stable group had the highest ADOS severity scores (6.8 ± 0.4 vs. 2.4 ± 0.1 in mid-high/high groups, χ²=96.81, p<0.001).	9/9 High
Leyan Li et al. (31)	Cross-lagged panel study examining motor and language trajectories in ASD-risk infants.	408 infants (271 elevated likelihood (EL; 46 later diagnosed with autism), 137 typical likelihood (TL))	ASD: SRS-2, MSEL Motor development: Vineland Adaptive Behavior Scales	- Autism Traitstraits (R²=0: Lower 24-month RL (β=-0.394, *p* =0.001) and 36-month GM (β=-0.253,.467)*p* =0.001) predicted higher SRS scores in EL group. GM-RL association explained 46.7% variance in autism. Explained 46.7% variance in autism traits (*R²* =0.467). - Group Differences: EL group had lower language/motor scores (e.g., EL at 36 months: EL group *M* =33.63 vs. TL group *M* =37.29, *p* < 0.001) and higher SRS scores (*M* =42.37 vs. 24.48, *p* < 0.001).	6/9 Moderate
Ben-Sasson et al. (32)	Longitudinal and Cluster Analysis of Developmental Milestone Trajectories in Autistic Children Using Electronic Health Records.	Experimental: 5,836 autistic children; no explicit control group.	K-means cluster analysis	- Cluster Differences. The global cluster had the earliest delays (71.64% passed “says one word” at 9–12 months vs. 0% by 36 months); the Severe cluster declined sharply at 18–24 months (40% passed “vocabulary >10 words”). The global cluster had the earliest delays (71.64% passed “says one word” at 9–12 months vs. 0% by 36 months); the Severe cluster declined sharply at 18–24 months (40% passed “vocabulary >10 words”). - Motor: Global cluster showed early gross motor delays (50% failure by 9–12 months); Severe cluster had later fine motor delays (46.13% passed “imitating lines” at 24%). Severe cluster had later fine motor delays (46.13% passed “imitating lines” at 24–36 months).	9/9 High
Elena Capelli et al. (33)	Longitudinal study exploring sensory-motor interplay and its link to autistic traits.	Experimental: 118 high-risk infant siblings; Control: No familial ASD history.	ASD : ADOS Motor development:GMDS-ER	- Significant within-domain stability: Sensory sensitivity (β=0.493, *p* < 0.001) and eye-hand coordination (β=0.357,.*p* < 0.001) from 12 to 18 months Lower eye-hand coordination at 12 months. - Lower eye-hand coordination at 12 months predicted higher sensory sensitivity at 18 months (β=-0.316, *p* < 0.001). - Sensory sensitivity (β=0.305, *p* < 0.001) and eye-hand coordination (β=-0.300, *p* < 0.001) at 18 months predicted autistic traits (CSS) at 24–36 months. CSS) at 24–36 months.	8/9 High
Wilson et al. (2024) (34)	Cross-sectional study on gait patterns and developmental measures in toddlers with ASD.	51 ASD toddlers and 45 typically developing toddlers, aged 12–36 months.	ASD : ADOS, Motor developmen:MSEL, VABS	Group Differences (ASD vs. TD). - Pace. - Velocity: ASD=0.89 cm/s vs. TD=1.13 cm/s (p < 0.001). - Cadence: ASD=1.78 steps/min vs. TD=2.17 steps/min (p < 0.001). - Step length: ASD=0.33 cm vs. TD=0.37 cm (p=0.002). - Postural control: No significant difference in stride width (p=0.99).	6/9 Moderate

**Figure 1:**
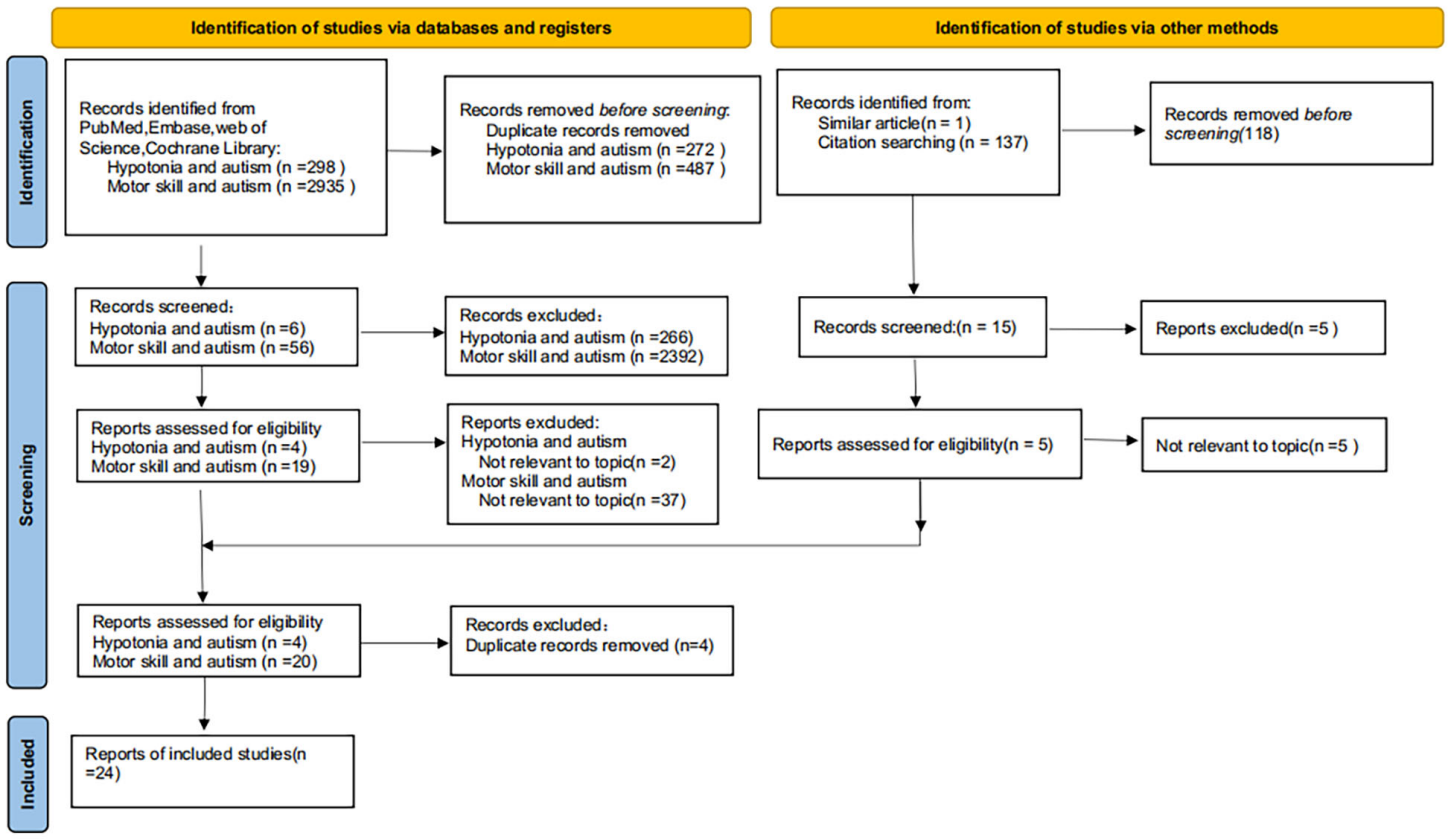
Flowchart of the study selection procedure.

The 24 studies had different study designs, including prospective cohort, case-control, and retrospective video analysis designs. The age of the participants varied from 2 months to 6 years. Study selection was performed with a focus on the correlation between hypotonia (encompassing motor delays) and autism. AMSTAR-2 was applied on all systematic reviews(A complete report of the risk of bias of the included studies is detailed in the **Supplementary Table 1**). Of the 24 studies evaluated, 14 were classified as high quality and 10 were deemed to be of moderate quality. The complete literature identification and screening procedure is illustrated in a flowchart by the PRISMA criteria (**Figure 1**).

### 3.2 Data items and data extraction

Two researchers (ZT and CZK) conducted data extraction and analysis using a dual-person verification system. In studies where hypotonia predicted autism, the following essential elements were systematically retrieved: study title, characteristics of the study population, study design type, intervention, assessment tool, principal findings (including effect sizes and confidence intervals), conclusion points, and quality of evidence rating. The data extraction approach for research on motor retardation for autism prediction was consistent with that used in previous frameworks. However, because of the significant heterogeneity of the included studies and the inadequate sample size, only descriptive summaries were generated, and no meta-analysis was performed. In the event of a disagreement during the investigation, consensus was achieved through a triadic collegial conversation with the senior investigator (WJY) to ensure the accuracy and consistency of data extraction.

### 3.3 Quality evidence assessment and risk of bias

The quality of evidence and risk of bias of the research included in this review were assessed by two independent evaluators (ZT and CZK). The quality of evidence from the original research was evaluated using the Newcastle-Ottawa Scale (35), whereas the risk of systematic bias was determined using the AMSTAR-2 criteria (36).

## 4 Overview of included studies

### 4.1 Potential for predicting autism in children displaying hypotonia

Several studies have examined the relationship between hypotonia (characterized by a diminished muscle tone) in infancy and autism in childhood, using multiple assessment methods to evaluate the impact. Notwithstanding methodological variations, these investigations have concentrated on the correlation between hypotonia and autism.

Yoshioka (22) reported that among children with hypotonia (floppy newborns) exhibiting delayed motor development during well-baby examinations, 27% were eventually diagnosed with autism. Subsequently, Serdarevic et al. (12) conducted a longitudinal study and observed a significant correlation between overall childhood hypotonia and scores on the Pervasive Developmental Problems Scale. This suggests that hypotonia increases the likelihood of autism and, in infants aged 2–5 months, could predict the potential for developing autistic traits at a later stage (approximately 6 years). Subsequently, in their follow-up study, Serdarevic et al. (27) found that higher autism polygenic scores were significantly associated with infant neuromotor differences, particularly hypotonia. Gabis et al. (11) conducted a study on 5,205 children and reported that children with autism and hypotonia received diagnoses significantly earlier—on average, 1.5 years earlier for males and 1 year earlier for females. We observed that term infants exhibited a particularly pronounced effect. López-Espejo et al. (10) conducted a cross-sectional observation of 96 children with autism. They found that 63.5% children exhibited motor impairments, including 28.1% with delayed gait (inability to walk independently by 16 months) and 32.3% with widespread hypokinesia. The study findings indicated that hypokinesia markedly elevated the incidence of gait delay (OR=2.65, 95% CI=1.08-6.48) and motor stereotypies (OR=2.63, 95% CI=1.04-6.65). This implies that hypokinesia may considerably impact motor development and general neurodevelopment in children with autism.

### 4.2 Correlation between conditions and autism-related mobility owing to hypotonia

Motor skills are essential for developing play, social interaction, communication, and language skills (37). Some children from the general population may exhibit neuromotor development issues (e.g., motor coordination and motor skills) that correlate with a later diagnosis of autism (38). Many of these neuromotor delays or difficulties, as developmental milestones, are linked to hypotonia (39, 40). Furthermore, these neuromotor developmental challenges may be present from birth in children with autism and represent a significant likelihood factor for autism (41). Although the connection between hypotonia and autism has not been investigated directly in most studies, the predictive value of hypotonia in autism diagnosis has been assessed.

Hypotonic abnormalities can coexist with these motor difficulties, including head lag, delayed development of fine motor skills, and potential gait delays. In this review, we used findings from multiple studies as indirect evidence, focusing major on the correlation between motor abilities and autism. In most of the selected studies, autism was evaluated using the Autism Diagnostic Observation Schedule (ADOS), a validated and standardized tool developed explicitly for autism diagnosis (42). In some studies, a combination of the Diagnostic and Statistical Manual of Mental Disorders (DSM) and ADOS was used, along with other assessment methodologies, whereas in a few studies, children with an elevated likelihood of a later autism diagnosis were assessed using questionnaires.

#### 4.2.1 Investigations highlighting the correlation between fine motor skills and autisms

Ten of the selected studies investigated the relationship between fine motor abilities and autisms. Six of them used the Mullen Scales of Early Learning (MSEL), a well-established tool for evaluating the developmental levels of infants and toddlers, especially with respect to fine motor skills (43).

Nordin et al. (29) identified that 38.5% of children with autism exhibited chronic fine motor delays in a retrospective study on 104 children with autism and 74 TD children aged 12 to 60 months, assessed using the SGS II scale (14, 17) conducted a prospective study showing that babies and children at elevated likelihood for autism exhibit a delay in fine-motor development between 6 and 14 months of age. The authors observed notable disparities even within high-likelihood groups that did not meet the clinical diagnostic criteria. Libertus et al. (20) demonstrated that, at 6 months, children in the high-likelihood group exhibited markedly lower proficiency in object manipulation during organized activities compared to children in the low-likelihood group and also exhibited a lower frequency of grabbing during free play.

Additional research indicated that diminished fine motor scores at 6 months correlate with an increased likelihood of autism later in life (15), and that ongoing motor delays are the most significant predictor of autism (28). Øien et al. (26) observed that this anomaly maintained discriminating validity within a routinely screening-negative cohort.

In a longitudinal study on 5,836 children with autism, Ben-Sasson et al. (32) showed that children with higher levels of autistic characteristics exhibited fine motor lag, an early indication of autism characteristics, and a progressive worsening of characteristics with age. Three studies have shown that fine motor lag directly or indirectly affects the trajectory of autism (24, 30, 33). The direct effects are evident in the predictive relationship between fine motor scores at 6 months and language abilities in children with autism at 3 years (24, 30), whereas the indirect effects influence core characteristics, such as repetitive, stereotyped behaviors, by augmenting sensory sensitivity (33).

#### 4.2.2 Research highlighting the correlation between gross motor skills and autisms

Nine studies investigated the correlation between gross motor skills and autisms. They primarily used standardized assessment instruments to measure the predictive significance of motor difficulties.

In a prospective follow-up, Flanagan et al. (16) discovered that high-likelihood infants at 36 months exhibited more head lag than low-likelihood children, suggesting that head lag may contribute to feeding issues in these infants. Furthermore, a substantial association was observed between feeding issues and autism. Infants displaying head lag typically exhibit poor coordination and reduced muscle tone in motor control (44). Infants diagnosed with autism in a study conducted by Sacrey et al. (25) exhibited significantly higher scores in reaching and grasping motions at 6–36 months than children from the low-likelihood and high-likelihood groups without an autism diagnosis.

Two studies investigated the correlation between gait retardation and autism, suggesting that retardation may serve as a potential genetic biomarker (23, 34). Bishop et al. (23) discovered that the mean age for independent walking in children with autism was 19 months, significantly later than that observed in TD toddlers. Furthermore, for each month of delayed independent walking, there was a 17% increase in the likelihood of the child possessing a *de novo* mutation. Conversely, Wilson et al. (34) found that children from the autism group had a significantly lower step speed, frequency, and length than children from the TD group, and that a lower step speed was associated with lower MSEL gross motor scores and Vineland Adaptive Behaviour Scale-II (VABS) adaptive functioning scores were significantly associated.

In four additional investigations, no specific motor behavior could be identified (18, 19, 21, 31). However, the findings consistently showed a substantial correlation between gross motor movements and autism development. Lemcke et al. (19) discovered that the “inability to sit upright on lap” at 6 months, “inability to drink independently” at 18 months, and “inability to climb stairs without support” were considerably more common in children from the autism group than in children from the TD group, and their incidence was markedly greater in the former group than in the latter.

Simultaneously, children with autism exhibited considerable delays in independent sitting and ambulation. Heathcock et al. (21) discovered that at 4 months, children in the high-likelihood group exhibited significantly lesser improvements in gross motor skills (AIMS scores) and upper extremity midline behavior than children in the low-likelihood group. LeBarton and Iverson (18) discovered that children from the autism group exhibited markedly lower rates of visual-motor integration skills, as measured using the Peabody Developmental Motor Scales, compared to children from the low-likelihood group. These assessment results were correlated with an autism diagnosis at 24–36 months. Li et al. (31) discovered a persistent correlation between language comprehension (RL) in the high-likelihood group and gross motor skills, with both 24-month RL and 36-month gross motor strongly predicting autism characteristics at 36 months.

## 5 Discussion

This systematic review synthesizes evidence from diverse study designs to evaluate the association between hypotonia in infancy and the subsequent likelihood of autism in childhood. The included studies encompassed both general pediatric populations and high-likelihood cohorts for autism, thereby enhancing the generalizability of the findings. The primary exposure of interest was hypotonia or related motor delays identified during infancy or early childhood, most commonly assessed via standardized scales or clinical evaluation (45). Control groups typically comprised children with typical development or high-likelihood children not diagnosed with autism. The majority of studies consistently indicated that early manifestations of hypotonia or marked motor delay often precede the core characteristics of autism by several months to years, and children exhibiting these phenotypes are at a higher likelihood of later autism diagnosis (46).

However, substantial heterogeneity was observed across studies in terms of participant selection, exposure definition, outcome measures, and methodological approaches. For instance, some studies focused on fine motor development, whereas others assessed gross motor milestones or head control. The variability in assessment tools and follow-up duration further limited the comparability of results. Moreover, although hypotonia is more prevalent among children with autism, it is not a feature unique to autism and may also occur in other neurodevelopmental disorders, suggesting that its utility as a clinical screening indicator must be considered within the context of multidimensional evaluation (47).

Despite a general consistency in findings, notable differences remain in terms of population inclusion criteria, definitions of exposure, outcome indicators, and methodological design. Some investigations targeted fine motor skills, while others emphasized gross motor milestones or head control, employing a range of assessment instruments and timeframes that impede direct comparison. Furthermore, the criteria used to define endpoints varied: some studies adopted formal autism diagnosis, while others assessed social, language, or adaptive functioning, and reported effect sizes and confidence intervals in differing manners.

Further analysis reveals that hypotonia is not only an early clinical manifestation of autism but may also reflect more profound neurodevelopmental abnormalities. Some neuroimaging and genetic studies have indicated that structural brain abnormalities associated with autism may be located in regions crucial for motor control, such as the frontal lobe, cerebellum, and basal ganglia (48–50). Atypical development in these areas may result in impaired muscle tone and motor coordination, which in turn can further disrupt social behavior and nonverbal communication abilities (41). Additionally, certain genes implicated in hypotonia overlap with those associated with autism, and disturbances in key inhibitory neurotransmitters—such as gamma-aminobutyric acid (GABA)—within the central nervous system are thought to contribute to both motor control difficulties and the exacerbation of autism characteristics (51, 52).

Indirect evidence also suggests that delays in gross motor skills (including postural control, motor acquisition, and limb coordination) and fine motor development (notably manual dexterity and visuomotor integration) are closely associated with autism onset in early childhood (15). Motor development delays can hinder children’s ability to explore their environment and impair visuomotor coordination, thereby disrupting the coordinated development of perceptual and motor systems, and exacerbating learning, cognitive, and social difficulties in children with autism (53, 54). Research indicates that children with autism may impact the psychological development of their siblings (55) and these high-risk children possess an 18.7% likelihood of developing autism again (56). This context underscores the importance of early vigilant monitoring of growth and development indicators (such as motor development and muscle tone) for younger siblings.

Although some studies did not designate hypotonia as a primary focus, their findings nevertheless provide valuable insights for the early identification of autism in high-likelihood infants and young children.For children with or at likelihood of autism, early identification and access to evidence-based, early intervention are critical.Evidence indicates that early intervention yields positive effects on communication, cognition, and adaptive functioning in children with autism (57). Autistic participants who receive early diagnosis accessed more intervention are more likely to attend mainstream schools, and require less ongoing support (58).

Given the potential association between hypotonia and autism, further research should examine the feasibility of using hypotonia as an early indicator of autism, thereby informing the refinement of intervention strategies and promoting improved quality of life and social inclusion for children with autism. Future investigations should prioritize the use of unified PICOS criteria and large-scale, prospective, multicenter clinical trials integrating genetic, neuroimaging, and neurophysiological measures, in order to elucidate the clinical and biological linkages between hypotonia and autism.

### 5.1 Clinical feasibility

This is the inaugural review assessing the significance of hypotonia as a predictor of autism, demonstrating how early hypotonia might indirectly influence or forecast autism by impacting motor performance. Despite evidence indicating that hypotonia could function as an early screening diagnostic for autism, there are obstacles to incorporating this indicator into standard clinical practice. Standardized motor assessment necessitates specific training for healthcare practitioners, and variations in assessment methodologies across environments may influence diagnostic consistency. Furthermore, early screening initiatives must reconcile feasibility with cost-effectiveness to guarantee that hypotonic evaluations may be incorporated into current pediatric examinations without imposing excessive resource demands. Future research should investigate the practicality of adopting hypotonic assessments across various cultural and socioeconomic contexts and how to optimize the assessment process under diverse resource constraints. Simultaneously, creating user-friendly and economical screening instruments is crucial for enhancing the accessibility and precision of hypotonia evaluation. This will assist help to optimize the prognosis for children with autism. Furthermore, creating an interdisciplinary collaborative framework to improve communication and cooperation across pediatrics, neurology, rehabilitation, and other disciplines is essential for enhancing the clinical feasibility of hypotonia assessment.

### 5.2 Limitations

Although this review provides valuable insights into the association between hypotonia and autism, the field is still in an exploratory phase with several limitations. First, the number of studies directly on the relationship between hypotonia and autism is small, especially in the infant and toddler populations with small sample sizes, and the lack of large-scale and broadly representative validation limits the generalizability of the findings. Second, the quality of studies varies widely, with some retrospective studies relying on historical records or parental recall, which tends to introduce recall bias, and cross-sectional studies failing to identify causal relationships. There was also heterogeneity in study methodology, study design, sample source, and assessment tools, which could quickly introduce selection bias, and the lack of a randomized control group further affected the reliability and reproducibility of the results and increased the difficulty in comparing the results. Although existing studies generally suggest that hypotonia is more common in children with autism, the neural mechanisms underlying it are unclear. Current evidence is insufficient to confirm whether hypotonia directly contributes to autism.

### 5.3 Recommendations for future research

Future research should prioritize longitudinal study designs to investigate the potential significance of hypotonia in the early screening of autism, mainly through long-term follow-up of high-likelihood groups during infancy. These study designs can systematically record the complete trajectory from birth to the diagnosis of autism, rigorously assess whether hypotonia is a reliable predictor, elucidate its causal relationship with autism development, and explore its underlying neural mechanisms.

Furthermore, the correlation between various manifestations of hypotonia and clinical characteristics of autism warrants investigation to establish a more scientific and accurate foundation for early screening and the formulation of intervention methods. Moreover, conventional autism diagnostic approaches predominantly depend on categorization criteria, potentially resulting in the misdiagnosis of persons with mild difficulties or those from multigenerational autistic families (38). Moreover, most contemporary research shows substantial variation in their evaluation instruments, and this methodological inconsistency may profoundly impact the comparability and trustworthiness of the results. Future research indicates the implementation of standardized quantitative systems to document the diversity of autism-related characteristics more precisely and accurately assess dystonic features and related motor developmental anomalies.

## 6 Conclusions

This review offers insight into the possible significance of hypotonia in the early screening of autism. Hypotonia and its related movement problems may function as practical screening markers to aid physicians in the early detection of high-likelihood youngsters and facilitate prompt therapies. Consequently, the viability of hypotonia as an early diagnostic instrument for autism necessitates additional research. Interdisciplinary collaboration and rigorous research may enable the prompt identification of children at elevated likelihood for autism through the early detection of hypotonia, resulting in earlier intervention and enhanced long-term outcomes.

## Data Availability

The original contributions presented in the study are included in the article/**Supplementary Material**. Further inquiries can be directed to the corresponding author.
