# Supplementary material for: Early muscle hypotonia as a potential marker for autism spectrum disorder: a systematic review
Source: Front Psychiatry. 2025 Sep 18;16:1598182. doi: 10.3389/fpsyt.2025.1598182 (PMC12488687; doi:10.3389/fpsyt.2025.1598182)
Supplement: Supplementary file 1 [file Table1.docx]

**Table 1:The risk of bias of the included studies**

|  | | Landa et al., (2006) | Bolton et al., (2012) | Flanagan et al. (2012) | Landa et al. (2013) | LeBarton et al,. (2019) | | Lemcke et al., (2013) | Libertus et al. (2014) | Heathcock et al., (2015) | Yoshioka et al., (2015) | Bishop et al. (2017) | Serdarevic et al. (2017) | Boin Choi et al., (2018) | | Sacrey et al., (2018) | Øien et al., (2018) | Serdarevic et al. (2020) | Gabis et al. (2021) | Licari et al., (2021) | Mohd Nordin et al., (2021) | López-Espejo et al. (2022) | | Patterson et al., (2022) | Leyan Li et al., (2023) | Ben-Sasson et al., (2024) | Elena Capelli et al., (2024) | Wilson et al., (2024) |
| --- | --- | --- | --- | --- | --- | --- | --- | --- | --- | --- | --- | --- | --- | --- | --- | --- | --- | --- | --- | --- | --- | --- | --- | --- | --- | --- | --- | --- |
| PICO included | |  |  |  |  |  | |  |  |  |  |  |  |  | |  |  |  |  |  |  |  | |  |  |  |  |  |
| Protocol registered | |  |  |  |  |  | |  |  |  |  |  |  |  | |  |  |  |  |  |  |  | |  |  |  |  |  |
| Selection of study designs explained | |  |  |  |  |  | |  |  |  |  |  |  |  | |  |  |  |  |  |  |  | |  |  |  |  |  |
| Adequacy of literature search | |  |  |  |  |  | |  |  |  |  |  |  |  | |  |  |  |  |  |  |  | |  |  |  |  |  |
| Study selection in duplicate | |  |  |  |  |  | |  |  |  |  |  |  |  | |  |  |  |  |  |  |  | |  |  |  |  |  |
| Data extraction in duplicate | |  |  |  |  |  | |  |  |  |  |  |  |  | |  |  |  |  |  |  |  | |  |  |  |  |  |
| Justification for excluding studies | |  |  |  |  |  | |  |  |  |  |  |  |  | |  |  |  |  |  |  |  | |  |  |  |  |  |
| Studies described in adequate detail | |  |  |  |  |  | |  |  |  |  |  |  |  | |  |  |  |  |  |  |  | |  |  |  |  |  |
| RoB assessment of individual studies | |  |  |  |  |  | |  |  |  |  |  |  |  | |  |  |  |  |  |  |  | |  |  |  |  |  |
| Reporting sources of funding for studies | |  |  |  |  |  | |  |  |  |  |  |  |  | |  |  |  |  |  |  |  | |  |  |  |  |  |
| Appropriateness of meta-analytical methods | |  |  |  |  |  | |  |  |  |  |  |  |  | |  |  |  |  |  |  |  | |  |  |  |  |  |
| Assessment of potential impact of RoB on results | |  |  |  |  |  | |  |  |  |  |  |  |  | |  |  |  |  |  |  |  | |  |  |  |  |  |
| Consideration of RoB in interpreting results | |  |  |  |  |  | |  |  |  |  |  |  |  | |  |  |  |  |  |  |  | |  |  |  |  |  |
| Heterogeneity explained and discussed | |  |  |  |  |  | |  |  |  |  |  |  |  | |  |  |  |  |  |  |  | |  |  |  |  |  |
| Assessment of publication bias | |  |  |  |  |  | |  |  |  |  |  |  |  | |  |  |  |  |  |  |  | |  |  |  |  |  |
| Conflicts of interest and review funding reported | |  |  |  |  |  | |  |  |  |  |  |  |  | |  |  |  |  |  |  |  | |  |  |  |  |  |
| Legend | yes： | | | | | | No: | | | | | | | | Partial Yes: | | | | | | | | Not Applicable: | | | | | |
